# Students’ Well-Being: The Mediating Roles of Grit and School Connectedness

**DOI:** 10.3389/fpsyg.2021.787861

**Published:** 2021-11-17

**Authors:** Kunni Han

**Affiliations:** ^1^College of Liberal Arts, Journalism and Communication, Qingdao University, Qingdao, China; ^2^Institute for Research on Portuguese-Speaking Countries, City University of Macau, Macau SAR, China

**Keywords:** grit, students’ well-being, teacher educators, school connectedness, positive psychology

## Abstract

A remarkable point in previous decades in every aspect of life is well-being which is also effective in academic settings, and it is consistent with positive psychology, in which one can recognize how to make everything pleasing. Moreover, grit is another noteworthy point in the process of learning, which is at the center of researchers’ attention in last years as a result of its long-term eminence. In addition, school connectedness is another important factor that was found to be positively related to students’ well-being. Therefore, the current review endeavors to emphasize the mediating role of these two constructs, grit and school connectedness on students’ well-being. Successively, some implications are proposed for educators, learners, teacher educators, and materials developers.

## Introduction

Positive Psychology (PP) has turned into a more popular issue in recent years, to encourage individuals to succeed and achieve (Lopez and Snyder, 2004; Wang et al., 2021) by focusing on the positive aspects of life (Csikszentmihalyi and Nakamura, 2011). The PP scholars cannot ignore the presence of difficulties, but primarily add the positive aspects namely energy, optimism, confidence, well-being, enthusiasm, imagination, pleasure, success, grit, endurance, good feelings, intelligence, kindness, self-respect, and sense of humor (Lopez and Snyder, 2004). Researchers are encouraged to examine emotions over a variety of timescales, such as near-term, second by second, as well as long-term, and years by year (MacIntyre and Mercer, 2014). The influence of well-being on life satisfaction has been identified by researchers using PP (Askell-Williams et al., 2013). According to the concept of subjective well-being (SWB), it is a degree of contentment among learners, which is individually determined based on their general satisfaction and other significant life aspects and their perceptions and feelings in terms of their well-being (Diener and Ryan, 2009). SWB has been characterized as the blend of significant degrees of life fulfillment, high self-announced positive affect, and low self-announced negative effect (Kansky and Diener, 2017).

Besides SWB, important outcomes have been linked to SWB in higher education, such as learning ambitions, educational participation, attending classes, choosing a field of study, learning success, and graduation (Nickerson et al., 2011; Xie and Derakhshan, 2021). So, not only enhancing learners’ SWB is an important factor, but also it is essential to educational and personal success. Indeed, a learner’s emotional connections are closely connected to their well-being. Social behavior and close relationships are common among those with high positive affect (Eid et al., 2003). For ideal functioning, and well-being, grounded on Self-determination theory (SDT), three distinctive psychological needs, such as autonomy, relatedness, and competence are indispensable (Ryan and Deci, 2000). Among these needs, autonomy support refers to how much the setting allows learners to feel independent and able to determine their decisions without interference (Grolnick, 2003). This autonomy support can be provided from different sources such as parents, teachers, peers, and schools and since learners principally spend their adolescence at schools, it has a notable function not only in the society but also in the scholastic progress of students (Carney et al., 2017).

Aside from character and positive mental orientations, contextual variables impact the healthy growth of adolescents (Parker et al., 2015). Specifically, school is a significant setting in teenagers’ day-to-day life, and their sense of connectedness to school impacts their perceived life quality, which is one of the aspects of well-being (You et al., 2008). Creating good connections at school and feeling associated with it are important for advancing positive scholarly results since it is an essential environmental setting for youths and their education (Furrer and Skinner, 2003). Learners who feel that they have received greater consideration and have a place inside the school experience more accomplishments and display less troublesome behaviors inside and outside of school (Brown and Evans, 2005). This idea is well-portrayed by school-connectedness as an indicator of instructive and societal results (Sampasa-Kanyinga et al., 2019) and it relates to how well learners can detect that they have a place inside schools or scholarly networks. Moreover, this construct encompasses learners’ impression of how they are valued in school settings (Townsend and McWhirter, 2005). As a positive factor of well-being, school connectedness refers to the learners’ feeling that educators and classmates (adults and peers) care about academic success and themselves as learners (Shochet et al., 2006). It corresponds with components like emotional well-being, scholastic motivation, and indexes of school performance (Kidger et al., 2012).

On the one hand, comprehensive exploration has discovered that school connectedness is a significant protecting issue for intellectual well-being and constructive youth growth (Lester et al., 2013) and it is related to learner’s participation in tasks, scholastic performance, and social development (Watson, 2017). On the other hand, poor school connectedness is related to high-risk manners (Govender et al., 2013). Learners are bound to be successful when they feel associated with their school since that emotion brings about versatility, instructive motivation, enhanced school participation, and fewer interactive issues (Ernestus et al., 2014). As declared by Davis (2006), along with this structure, teenagers who take in a positive school climate and constructive associations with educators have higher grades, accomplishment test scores, scholarly self-efficacy, and school engagement. School connectedness has been connected to a scope of positive scholastic results, including learners’ commitment, scholastic accomplishment, achievement prospects, self-efficacy, endeavor, and scholarly enthusiasm (Witherspoon et al., 2009; Niehaus et al., 2016).

Furthermore, another distinctive feature that is deemed as a part of disposition and consequently significant for an individual’s life to grasp his/her aims is grit (Akbag and Ümmet, 2017) and there is an increasing review of literature presenting its effect on well-being. Grit is considered by supporters as a good sign of educational and professional success across a wide array of careers (Duckworth et al., 2007; Maddi et al., 2012). Grit is divided into two factors: persistence of interest, a tendency to continue activities over a long time, and commitment of effort, a willingness to overcome challenges and persevere until success is achieved (Duckworth et al., 2011). Learners’ performance and decisions in a variety of contexts are influenced by grit, which is defined as a personal characteristic (Reed et al., 2012). It becomes apparent as one grows and it can be promoted by developing passions, learning techniques, having a direction in life, and creating optimism (Duckworth, 2016). Students with more grit in academic situations are more likely to practice systematically and diligently, exhibit higher attendance rates, and are less likely to switch studies or fields. Additionally, grit tends to induce higher commitments, greater feelings of community, greater participation in extra-curricular activities and improves communication with educators (Bowman et al., 2015). It also produces different teaching methods that are less enjoyable to deal with complex problems (Duckworth et al., 2011). In particular, more gritted students stated that they are more likely to use their aptitudes as they are less motivated by temporary purposes and are less frustrated by obstacles and challenges associated with many types of performance (Credé et al., 2017). Based on the above-mentioned points and their significant effect on learning, the present review makes efforts to take grit and school connectedness together and examine how these issues can work as mediating functions on learners’ well-being.

### Grit

As stated by Duckworth (2016), grit as a cognitive trait has been predicted to be a tool for accomplishment in a wide variety of fields. Thus, grit has attracted significant interest among researchers. Persistence was originally conceived as a character strength, while being grouped with others by Seligman (2002). Grit has been extended, elaborated, popularized, and established by Duckworth et al. (2007). The capability to persist to accomplish a task despite the obstacles that may appear is tied to grit (Sturman and Zappala-Piemme, 2017). Duckworth presented the construct of grit, as passion and perseverance for long-term objectives (Duckworth et al., 2007). However, the persistence of passion did not guarantee success in educational performance or well-being, although dedication of effort was linked to these (Datu et al., 2017). Furthermore, across individuals throughout the world, the persistence of effort was associated with enhanced well-being and personal qualities, whereas persistence of interest, by contrast, was not positively related to these factors (Disabato et al., 2019). In collectivist settings, being consistent might not be important for learners, since continuously reflecting on individual beliefs and values in different circumstances can disrupt healthy personal interactions (Suh, 2007). Perseverance of effort requires endurance in difficult circumstances to accomplish long-term objectives (Duckworth et al., 2007), while flexibility is considered as adjusting to changes in a process or objective-associated strategy based on context or environmental issues.

According to Hogan and Wong (2013), gritty learners often put in more effort, persevere, and participate in intentional activities to improve efficiency and achievement. Learners have limitations in terms of performance due to their inherent talents and capabilities and must strive to improve certain aspects of achievement on their own (Duckworth et al., 2011). Learners who are more confident and thoughtful are less frustrated by challenges or obstacles, more attentive to their goals, and more likely to succeed in performing duties and the correlation between grit and wellbeing as intensely influenced by the extent to which a person perceives their world to be meaningful and manageable (Arya and Lal, 2018). Contrary to this, learners with poor gritty tend to be less committed and hard-working, susceptible to distraction by new opportunities, incapable of setting long-term goals, as well as lacking enthusiasm and commitment for long-term projects (Bazelais et al., 2016).

### Well-Being

Individuals’ experiencing fewer negative emotions, more frequent positive emotions, and more fulfillment in their lives can be known as SWB (Diener, 2012). As it can be inferred from this definition, SWB has two aspects, namely, intellectual and emotional. While individuals’ judgment concerning their life fulfillment is the intellectual aspect of SWB, known as satisfaction (Dorahy et al., 2000), the emotive aspect considers both the constructive and destructive emotions (Rask et al., 2002). SWB is thoroughly connected to how an individual assesses his/her own life regarding emotions and intellect (Diener et al., 2003). Thus, an individual’s SWB is characterized by his/her internal proficiencies and estimated by his/her point of view. Diener and Ryan (2009) asserted that individuals with developed SWB are inclined to be more inspired, diligent, optimistic, and supportive; moreover, they live longer and their inclination of being egotistical and antagonistic is uncommon. Roberts (2009) mentioned that individuals who have high degrees of grit are effortlessly motivated for objective-oriented practices and resilient attributes in an objective-oriented disposition. For an individual to accomplish his/her objective, these qualities are viewed as the most crucial bases and as stated by Rajabi and Ghezelsefloo (2020), some issue such as job stress, anxiety, and depression can reduce the level of well- being. It can be summarized from these definitions that are possible for an individual who has a grit character quality to accomplish his/her objectives (Vainio and Daukantaitė, 2016). Therefore, individuals who accomplish their objectives with their grit practices are estimated to enhance their well-being.

### School Connectedness

School connectedness is broadly connected to school-based adaptive results during puberty (Ciani et al., 2010). The cheerful, agreeable encounters of school connectedness have been decidedly associated with youths’ motivated conduct, self-concept, scholarly achievement, improved social and emotional growth, and well-being (Cook et al., 2012). The absence of school connectedness might result in failure, low scholarly performance, high-risk practices, and weak psychological well-being (Cook et al., 2012). School connectedness is broadly associated with school-based adaptive results during puberty. The cheerful, agreeable encounters of school connectedness have been decidedly associated with youths’ motivated conduct, self-concept, scholarly achievement, improved social and emotional growth, and well-being (Walton and Cohen, 2011; Cook et al., 2012).

Regularly situated in talks of “school connectedness” are connections, both inside the school and toward instruction. Constant evidence demonstrates that “school connectedness” is associated with a constructive feeling of well-being (Rowe et al., 2007). It has been proposed, however, that as much as most of all school learners have a challenging feeling of school connectedness (Sulkowski et al., 2012), mainly due to differences with the overall “logic of education.” This can prompt learners’ withdrawal and tension with educators, reinforcing an absence of connectedness and affecting well-being (Patton et al., 2000). College graduates are known to possess greater grit than some undergraduates who did not finish college (Duckworth et al., 2007). Grittier learners might be better at managing their learning and tracking their progress toward completing or succeeding in a course.

## Implications and Future Directions

School connectedness and grit are significant concepts in the field of instruction. Studies have demonstrated that positive associations with educators can forestall or diminish teenagers’ involvement in risk practices (Bonell et al., 2007). Therefore, educators and administrators should consider the results of the research if they wish to improve learners’ well-being. The educational atmosphere has a significant impact on a learner’s well-being, so it makes sense for educational institutions to use methods that are likely to enhance learners’ engagement. Highly gritty people are especially prone to be encouraged to look for engagement. Based on the methods of happiness theory, individuals are motivated to seek well-being through engagement, significance, or delight. Von Culin et al. (2014) asserted that people with high degrees of grit were less probable to seek delight, and they proposed this was because of the transient quality of pleasurable encounters, which greatly opposes the long-term engagement of gritty people. According to SDT, social conditions that provide people chances to fulfill the three previously mentioned essential demands will cultivate enthusiasm and mental well-being (Ryan and Deci, 2000). Hence, a school climate that fulfills learners’ demands for relatedness, capability, and independence will improve their well-being. A strong feeling of belonging in the school resulted from close communications with educators, staff fellows, supervisors, cleaning staff, and teaching assistants.

This review can be insightful for learners since grit is an important quality as it allows learners to accomplish their objectives in a way that fits their personalities. Indeed, the traits of grit in students are essential for making wise judgments and the positive outlook of grit might give individuals a sense of motivation, which in turn might encourage them to pursue their goals. Having grit in a decision, performing it, and achieving their targets, is seen as having a positive effect on individual well-being which is determined by the quality of their lives. Concerning the effect of grit on well-being, it can be stated that gritty people demonstrate consistent and persistent behavior to acquire long-term goals in their lives and can easily manage their emotions in case of facing obstacles (Duckworth et al., 2007; Roberts, 2009). Learners with gritty characteristics are persistent and flexible when faced with challenges (Bailly et al., 2012). Furthermore, they perform well under pressure and are not discouraged quickly. This makes them better equipped and capable of meeting their autonomy, expertise, and relationship needs. Consequently, their sense of well-being is significantly improved. Nevertheless, further experimental data are necessary to prove this conclusion. A receptive attitude toward their academic regulations also supported their sense of belonging to the institution as well as provided chances for making a significant improvement to their education (Yuen et al., 2012). The lack of safety in classrooms makes learners feel disconnected from their education, which reduces their motivation to pay attention or participate in academics, which adversely influences their educational performance. An improved sense of school connectedness could result in a positive impact on overall well-being and acts as protection contrary to emotional and psychological health issues in the future.

Regarding teachers, a comprehensive school counseling service should provide educators with relevant resources for educational enhancement so that they are equipped with the necessary expertise and abilities to regard the specific desires of learners in academic, psychological, and social domains. While at most colleges, advisor-to-learner relations are growing, it makes it more difficult for the college counselor to promote academic connectedness among all learners. Educators and school staff can also work with educational psychologists to develop effective learning communities that promote learner engagement. Interestingly, one of the main aspects of the autonomous theory that supports the requirement for relationship or connection is the interaction between connectedness in education and interdependence (Ryan and Deci, 2000). According to SDT, relatedness provides an important mechanism for integrating behaviors that are derived from extrinsic motivation and making them more autonomous. In this case, school officials who satisfy learners’ need for relatedness can assist them in feeling more associated with their school members. The result will be better motivation for learners to conduct effective academic behavior. The academic connectedness of learners has been reported to lead to improved performance in class and reduce disruptions in the classroom (Jdaitawi, 2015). Also, to raise emotional well-being for higher education and other educational levels, intervention programs can be developed by material developers along with strategies designed to maximize learners’ grit and comfort with their basic personal needs (Duckworth, 2016). Finally, to examine how school connectedness can be intensified, further research, principally experimental ones should be done in this regard.

## Author Contributions

The author confirms being the sole contributor of this work and has approved it for publication.

## Conflict of Interest

The author declares that the research was conducted in the absence of any commercial or financial relationships that could be construed as a potential conflict of interest.

## Publisher’s Note

All claims expressed in this article are solely those of the authors and do not necessarily represent those of their affiliated organizations, or those of the publisher, the editors and the reviewers. Any product that may be evaluated in this article, or claim that may be made by its manufacturer, is not guaranteed or endorsed by the publisher.
